# Dysautonomia and dysosmia associated with anti-Hu antibodies induced by immune checkpoint inhibitors: a case report

**DOI:** 10.1186/s12883-026-04880-y

**Published:** 2026-04-22

**Authors:** Masataka Fujimoto, Kota Igari, Motoki Fujimaki, Shinji Saiki

**Affiliations:** 1 School of Medicine, Faculty of Medicine, University of Tsukuba, 1-1-1 Tennodai, Tsukuba, Ibaraki 305-8575 Japan; 2 Department of Neurology, University of Tsukuba, 1-1-1 Tennodai, Tsukuba, Ibaraki 305-8575 Japan

**Keywords:** Immune checkpoint inhibitors, Dysautonomia, Anti-Hu antibodies, Autoimmune encephalitis, Dysosmia, Neurological immune-related adverse events

## Abstract

**Background:**

The use of immune checkpoint inhibitors may cause neurological immune-related adverse events in patients. Here, we report a case of autonomic nervous system involvement in nivolumab-induced autoimmune encephalitis with dysautonomia.

**Case presentation:**

A 78-year-old man presented with a 4-month history of orthostatic hypotension, dysgeusia, and dysosmia following a 2-month course of nivolumab prescribed for malignant mesothelioma. Serum was positive for anti-neuronal nuclear antibody type 1. Diagnostic work-up, including imaging, neurological, and biochemical tests, suggested the diagnosis of nivolumab-induced autoimmune encephalitis with pronounced autonomic nervous system involvement. On day 10 of hospitalization, the patient received high-dose intravenous methylprednisolone followed by oral prednisone. The patient was discharged on day 29 with continued administration of corticosteroids and midodrine. At the 1-month outpatient follow-up, the patient remained stable and reported independent ambulation without marked orthostatic symptoms.

**Conclusions:**

We discuss a possible mechanism of dysautonomia in this case and draw attention to newly emerging autonomic symptoms and olfactory impairments during therapy with immune checkpoint inhibitors.

## Key points

What is already known on this topic:

• Neurological immune-related adverse events occasionally occur in patients treated with immune checkpoint inhibitors.

What this study adds:

• Autonomic nervous system disturbances, dysgeusia, and dysosmia may be potential manifestations of neurological immune-related adverse events associated with positivity for anti-Hu antibodies.

How this study might affect research, practice or policy:

• Orthostatic hypotension following treatment with immune checkpoint inhibitors can be improved by treatment with corticosteroids and midodrine.

• To ascertain the diagnosis in such cases, diagnostic workup should include a broad spectrum of modalities, such as magnetic resonance imaging, cognitive and neurological tests, as well as measurements of plasma norepinephrine and arginine vasopressin levels.

## Background

Neurological immune-related adverse events (irAEs) occur in 1–5% of patients receiving immune checkpoint inhibitors (ICIs) [1]. Common neurological irAEs include myositis and myasthenia gravis, followed by peripheral neuropathy, encephalitis, cranial nerve involvement, and meningitis [2]. However, the involvement of the autonomic nervous system, dysgeusia, and dysosmia remain relatively rare. Furthermore, possible mechanisms and lesion sites underlying autonomic dysfunctions in patients with such manifestations are unclear. Here, we describe potential autonomic nervous system involvement in a patient diagnosed with nivolumab-induced autoimmune encephalitis with prominent dysautonomia. Specifically, we explored the association of clinical signs with anti-neuronal nuclear antibody type 1 (also known as anti-Hu antibody). Our findings emphasise the importance of recognizing autonomic dysfunction and dysosmia in patients receiving ICIs to enable early diagnosis and appropriate treatment.

### Case presentation

A 78-year-old man presented with a 4-month history of orthostatic hypotension, dysgeusia, and dysosmia. Six years earlier, he had undergone resection of a malignant mesothelioma of the left scrotum, followed by chemotherapy with cisplatin and pemetrexed. Pelvic lymph node resection was performed 5 years earlier, and computed tomography (CT) confirmed inguinal lymph node involvement 2 years earlier, which was followed by cisplatin-based chemotherapy. He received nivolumab 240 mg every 2 weeks for 2 months before neurological symptom onset, and nivolumab was stopped 1 month after onset.

Physical findings did not indicate dry mouth or dry eyes. Neurological examination revealed a Mini-Mental State Examination score of 23 of 30 and Frontal Assessment Battery score of 13 of 18. The patient reported no subjective abnormalities in sweating or upper gastrointestinal function, and no significant urinary disturbance. However, the patient experienced constipation and exhibited proprioceptive dysfunction in the lower extremities, decreased vibration sensation in the lower limbs, decomposition in the left heel-to-shin test, slight bilateral terminal tremor in the finger-to-nose test, and wide-based gait. Pain sensation was normal, and deep tendon reflex was preserved. No abnormalities in consciousness, ocular movement, nystagmus, pathological reflexes, or parkinsonism, including akinesia, tremor, or rigidity, were observed.

The Schellong test revealed a marked orthostatic decrease in blood pressure from 100/60 mmHg in the supine position to 68/45 mmHg, with a slight increase in heart rate from 86 to 93 bpm. immediately after standing. Plasma norepinephrine (NE) and arginine vasopressin (AVP) levels increased from 241 to 1.3 pg/mL in the supine position to 530 and 3.5 pg/mL upon standing, respectively, suggesting preganglionic autonomic dysfunction. Other specific tests assessing autonomic function, including sweating, gastrointestinal motility, and bladder function, were not performed. Electrogustometry demonstrated severe bilateral dysgeusia with thresholds > 34 dB in the chorda tympani, glossopharynx, and greater petrosal nerves. Olfactory testing revealed a significantly elevated recognition threshold of 5.8, indicating severe hyposmia.

Complete blood count, biochemical and coagulation profiles, vitamin B concentrations, and tumor markers (carcinoembryonic antigen, carbohydrate antigen 19 − 9, and cancer antigen 125) were normal. However, the autoimmune serology test was positive for anti-Sjögren’s syndrome-related antigen A autoantibodies (48.5 U/mL) and negative for autoantibodies against the ganglionic acetylcholine receptor. Serum testing using the autoimmune encephalitis panel (Mayo Clinic) yielded a positive result for anti-Hu antibodies (immunoblot positive; tissue-based immunofluorescence assay reactive with a titer of 1:61440). Cerebrospinal fluid (CSF) parameters were unremarkable, with a normal immunoglobin G index, absence of oligoclonal bands, negative cytology, sterile bacterial culture, and negative polymerase chain reaction results for John Cunningham virus. Antibody testing in CSF for autoimmune encephalitis was not performed. Chest and abdominal CT to screen for other tumors provided no evidence for additional lesions, such as lung cancer, beyond the known malignant mesothelioma.

**Fig. 1:**
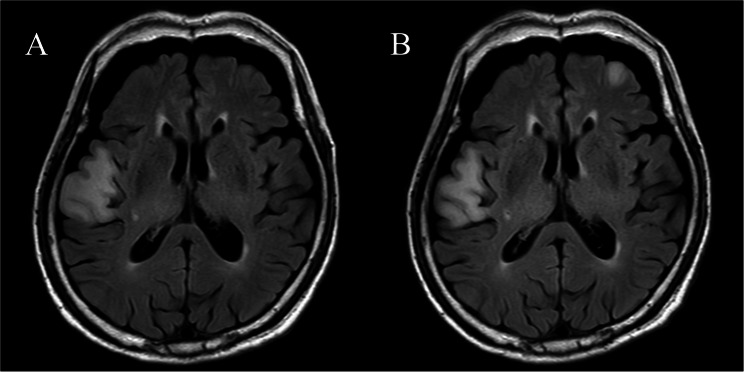
**A **Axial fluid-attenuated inversion recovery image showing a hyperintense lesion in the right frontal region around the Sylvian fissure, measuring approximately 34 mm in maximal diameter. **B **Axial fluid-attenuated inversion recovery image obtained 2 weeks after the image in (**A**), showing persistence of the lesion in the right frontal region and a newly emerged hyperintense lesion in the left frontal lobe, measuring approximately 13 mm in maximal diameter

Magnetic resonance imaging (MRI) of the brain revealed a hyperintense lesion in the right frontal cortex and white matter on a fluid-attenuated inversion recovery (FLAIR) scan without enhancement (Fig. 1) and on a contrast-enhanced T1-weighted image.

A follow-up FLAIR scan 2 weeks later showed a new white matter lesion in the left frontal lobe (Fig. 1B). ^123^I-metaiodobenzylguanidine (MIBG) myocardial scintigraphy showed a heart-to-mediastinum (H/M) ratio of 2.02 and 1.80 (normal > 2.2) in the early and late phases, respectively. Peripheral nerve conduction measurements demonstrated small right sural sensory nerve action potentials, whereas other nerves showed preserved action potential amplitudes and conduction velocities.

We considered two possible diagnoses: (1) neurological syndromes possibly associated with anti-Hu antibodies induced by ICIs and (2) clinically silent paraneoplastic neurological syndromes (PNS) secondary to mesothelioma enhanced by ICIs. The total score was 7 points, fulfilling probable PNS criteria: clinical level—high-risk phenotype, 3 points; laboratory level—high-risk antibody (anti-Hu), 3 points; and cancer consistency—not consistent with the antibody profile with follow-up < 2 years, 1 point [3]. In rare cases of anti-Ma2-positive PNS associated with mesothelioma, patients typically presented with opsoclonus, cerebellar signs, or conjugate gaze palsy [4], which differed from the clinical manifestations in our patient. Additionally, anti-Hu antibodies are commonly associated with lung cancer, endocrine tumors, and neuroblastoma. To the best of our knowledge, anti-Hu-positive PNS owing to mesothelioma has not been reported previously. Given the timing of ICI administration and absence of findings suggestive of infection or malignancy, the patient was diagnosed with nivolumab-induced autoimmune encephalitis with prominent dysautonomia.

High-dose intravenous methylprednisolone (1 g/day for 3 days) was initiated on day 10 of hospitalization, and oral prednisone 25 mg/day was administered after the first pulse. The second course commenced on day 18 owing to ongoing orthostatic symptoms and gait instability. Oral midodrine was initiated at 7.5 mg/day, resulting in a subjective reduction in dizziness upon standing. The patient was discharged on day 29 with continued administration of corticosteroids and midodrine. At the 1-month outpatient follow-up, he reported further stabilization and independent ambulation without significant orthostatic symptoms. The Schellong test demonstrated markedly improved orthostatic hypotension, as blood pressure changed only slightly: from 113/67 mmHg in the supine position to 102/73 mmHg immediately after standing. However, olfactory testing confirmed persistent dysosmia with thresholds comparable to those observed in the previous evaluation.

## Discussion and conclusions

Previous reports on ICI-associated dysautonomia described various clinical presentations, including orthostatic hypotension, gastrointestinal dysmotility, and urinary retention [5]. Tezuka et al. attributed dysautonomia to autoimmune autonomic ganglionopathy (AAG) or autonomic neuropathy [5]. All three patients in their study tested negative for anti-ganglionic acetylcholine receptor (gAChR) antibodies, which are important serological markers of AAG, and paraneoplastic antibodies. Similarly, our patient tested negative for anti-gAChR antibodies.

In our study, ^123^I-MIBG myocardial scintigraphy revealed a decreased H/M ratio in the early and delayed phases, suggesting impaired postganglionic sympathetic innervation. In contrast, the patient’s plasma NE level was normal in the supine position, and both NE and AVP levels increased upon standing, which may indicate the involvement of preganglionic fibers or central autonomic pathways. Both the autonomic ganglia and central autonomic network were considered to be involved in autonomic failure, although the precise lesion site could not be clearly identified. To the best of our knowledge, plasma NE and AVP levels were rarely measured in previous reports on ICI-related dysautonomia. Our case offers additional insights by combining these measurements with clinical findings to support the diagnosis of multilevel autonomic involvement.

ICI-induced immune activation may trigger responses against neuronal antigens, damaging neurons. However, dysosmia has rarely been reported as an adverse event associated with ICIs. Neurological irAEs include cranial nerve neuropathies, and several reports have described olfactory dysfunction in autoimmune encephalitis [6]. These findings suggest that dysosmia is underdiagnosed in patients with irAEs, necessitating further studies to clarify its prevalence and underlying mechanisms.

Malignant mesotheliomas are not commonly associated with anti-Hu antibodies; therefore, PNS was considered unlikely. The most common clinical feature of anti-Hu antibody-associated paraneoplastic syndromes is sensory neuropathy. Other neurological manifestations include cerebellar ataxia, limbic or brainstem encephalitis, and autonomic brain dysfunction [7, 8]. Approximately half of all patients with ICI-related autoimmune encephalitis harbor onconeural antibodies, particularly anti-Hu antibodies [9]. Although such antibodies are often associated with malignancies, particularly small-cell lung cancer, they can be detected as part of the irAE spectrum, independent of tumor progression. Therefore, anti-Hu antibodies induced through immune activation by ICIs might have partially contributed to the symptomatology in this case.

Furthermore, anti-Hu-positive encephalitis after ICIs is characteristically severe and often fatal. In a center-based cohort of 11 patients with anti-Hu antibody neurological irAEs, severe neurological disability at presentation was common (modified Rankin Scale > 3 in 91%), and despite therapies—corticosteroids, intravenous immunoglobulin, plasma exchange, cyclophosphamide, rituximab, and tocilizumab—no patient achieved sustained clinical improvement, and only one showed a transient response before rapidly worsening. Overall mortality reached 91%, with 5 of 10 deaths attributed to neurologic toxicity [10]. Clinical symptoms in four patients included respiratory insufficiency, and such severe neurological deficit likely contributed to mortality. Brain MRI frequently demonstrated inflammatory lesions, including limbic encephalitis (67%), medial temporal involvement (40%), and brainstem lesions (20%).

In contrast to these typical trajectories, our patient did not develop life-threatening features such as respiratory failure or limbic and/or brainstem encephalitis. Owing to the differences in clinical presentation and lesion localization compared with those in previously reported cases of anti-Hu antibody neurological irAEs, our patient followed a relatively mild, gradually improving course that did not require escalation beyond high-dose steroids and symptomatic management. These findings suggest that broader irAE-associated mechanisms induced by ICI therapy might have been involved in this case. Although published reports of ICI-related hypotension are limited, management has generally included vasopressors with immunotherapy [5]. In our patient, orthostatic hypotension improved after treatment with corticosteroids and midodrine. These observations suggest that ICI-associated hypotension does not necessarily cause severe, refractory symptoms and can often be controlled; however, the optimal agent remains uncertain.

In conclusion, our case highlighted a possible mechanism of dysautonomia and the importance of recognizing autonomic dysfunction and dysosmia as potential manifestations of neurological irAEs associated with positivity for anti-Hu antibodies. Clinicians must remain vigilant of newly emerging autonomic symptoms and olfactory impairments during ICI therapy. Detailed descriptions of future similar cases and mechanistic studies are needed to clarify the full spectrum of anti-Hu antibody-associated presentations following ICI therapy.

## Data Availability

Data sharing is not applicable to this article as no datasets were generated or analyzed during the current study.

## Abbreviations

AAG: Autoimmune autonomic ganglionopathy
AVP: Arginine vasopressin
CSF: Cerebrospinal fluid
CT: Computed tomography
FLAIR: Fluid-attenuated inversion recovery
gAChR: Ganglionic acetylcholine receptor
H/M: Heart-to-mediastinum
ICIs: Immune checkpoint inhibitors
irAEs: Neurological immune-related adverse events
MIGB: Metaiodobenzylguanidine
MRI: Magnetic resonance imaging
NE: Norepinephrine
PNS: Paraneoplastic neurological syndromes
